# Unsafe abortion requiring hospital admission in the Eastern Highlands of Papua New Guinea - a descriptive study of women’s and health care workers’ experiences

**DOI:** 10.1186/s12978-015-0015-x

**Published:** 2015-03-21

**Authors:** Lisa M Vallely, Primrose Homiehombo, Angela Kelly-Hanku, Andrea Whittaker

**Affiliations:** Australian Centre of Tropical Medicine and Health, James Cook University, Townsville, QLD Australia; Sexual & Reproductive Health Unit, Papua New Guinea Institute of Medical Research, PO Box 60, Goroka, 441 Eastern Highlands Province Papua New Guinea; School of Public Health and Community Medicine, UNSW Australia, Sydney, Australia; School of Political and Social Inquiry, Faculty of Arts, Monash University, Melbourne, Australia

**Keywords:** Unsafe abortion, Abortion methods, Misoprostol, Traditional herbs, Papua New Guinea

## Abstract

**Background:**

In Papua New Guinea induced abortion is restricted under the Criminal Code Law. Unsafe abortions are known to be widely practiced and sepsis due to unsafe abortion is a leading cause of maternal mortality.

**Methods:**

We undertook a six month, prospective, mixed methods study at the Eastern Highlands Provincial Hospital. Semi structured and in depth interviews were undertaken with women presenting following induced abortion. This paper describes the reasons why women resorted to unsafe abortion, the techniques used, decision to seek post abortion care and women’s reflections post abortion.

**Results:**

28 women were admitted to hospital following an induced abortion. Reasons for inducing an abortion included: wanting to continue with studies, relationship problems and socio-cultural factors. Misoprostol was the most frequently used method to end the pregnancy. Physical and mechanical means, traditional herbs and spiritual beliefs were also reported. Women sought care post abortion due to excessive vaginal bleeding, and severe abdominal pain with some afraid they would die if they did not seek help.

**Conclusion:**

In the absence of contraceptive information and services to avoid, postpone or space pregnancies, women in this setting are resorting to unsafe means to end an unwanted pregnancy, putting their lives at risk. Women need access to safe, effective means of abortion.

## Background

Of the 44 million abortions that took place globally in 2008 nearly half were considered unsafe [1], undertaken either by individuals without the necessary skills to perform the procedure, or were self-induced [2]. Forty percent of women seeking induced abortion live in countries where it is legally restricted. But even where induced abortion is legal, access to such services is often poor [3]. Most unsafe abortions occur in developing countries, in settings where standards of care are often poorer and legal restrictions are greater [2-4]. Every year an estimated 47,000 women die and five million women are hospitalized due to complications from unsafe abortions [2,3].

Methods of unsafe abortion include: the ingestion of harmful substances, physical means such as insertion of a foreign object or substance through the cervix and into the uterus, and external force, such as squeezing or massaging the abdomen [2,4-6]. It is suggested that the increasing availability and clandestine use of the E1 prostaglandin analogue, misoprostol is replacing many of these riskier methods of unsafe abortion in a number of countries [4,7,8]. In developing countries, severe complications and maternal deaths are lower with the use of misoprostol, even when used incorrectly, when compared to physical means of unsafe induced abortion [9,10].

Induced abortion is a sensitive issue, attracting moral condemnation, with those implicated in its practice frequently stigmatised [11]. Stigma may be perceived or experienced for those seeking both abortion and post abortion care; stigma is also recognised in relation to service delivery and at the policy level [12,13]. In countries where induced abortion is restricted by criminal law or inaccessible due to socio-cultural or geographical barriers, seeking information on incidence, practices and outcomes related to induced abortion is difficult. When it occurs in clandestine situations, abortion may not be reported or declared as a spontaneous abortion, due to stigma or barriers such as fear of prosecution [12].

### The situation in PNG

Papua New Guinea (PNG) is a low-middle income, developing country [14,15] situated in the Asia-Pacific region. It is a country notable for its socio-cultural and linguistic diversity. Eighty seven percent (87%) of the 7.2 million people reside in rural and remote areas with poor transportation. While 61% of the population are in paid employment it is notable that the majority of those residing in the rural settings remain as subsistence farmers [16,17]. Tribal conflict and accusations of sorcery between different communities or language groups remains common in PNG and poor security affects access to services in many areas. High rates of domestic violence and rape are also reported [18-22].

Maternal health indicators in PNG are poor. The country has a low contraceptive prevalence rate for modern methods of family planning among married women (24%) and a high unmet need for family planning (27%) [23,24]. The maternal mortality ratio is the highest in the Oceania region and one of the highest in the world, with an estimated 594 maternal deaths per 100,000 live-births [25]. Puerperal sepsis and sepsis due to unsafe abortion are reported as the second leading cause of maternal mortality, after post-partum haemorrhage [24,26].

In PNG, induced abortion to save a woman’s life or to preserve her physical and mental health may be granted on agreement by two medical officers. However, virtually no safe abortions take place in government facilities throughout the country. Under PNG’s Criminal Code Act, abortion for socio-cultural reasons or on request remains illegal [27]. Despite the criminal law surrounding abortion, induced, unsafe abortions are known to be practised, although documented evidence is limited. Traditional, herbal abortifacients and physical and mechanical means to end an unwanted pregnancy are described from a number of societies within PNG [19,28,29]. Self-starvation, self-poisoning, avoidance of antenatal care, and the use of traditional and modern contraceptives, such as the “morning after pill” to terminate an unwanted pregnancy are reported [19]. More recently, as part of a wider behavioural surveillance survey undertaken in Port Moresby, reports of unsafe abortion included the use of herbal medicines and “drinking tablets” [30]. The exact nature of the tablets was not reported.

Aside from earlier work surrounding sexual and reproductive health that highlighted women’s experiences of induced abortion, [19] no study has described women’s experiences of induced abortion, specifically relating to the socio-cultural context within PNG. The overall aim of this paper is to describe, from one setting in PNG, the reasons why women resort to unsafe abortion, the techniques used, the consequences leading to hospital admission and the reasons behind both the abortion and seeking post abortion care.

## Methods

As part of a prospective, mixed-methods study we undertook case note review, semi-structured and in-depth interviews with women admitted to hospital for post abortion care. We also undertook in depth, key informant interviews with health care professionals. Data collection took place over a six month period between May and November 2012 at the Eastern Highlands Provincial hospital, Goroka, Eastern Highlands Province, Papua New Guinea. All data collection, including clinical data and interviews was undertaken by one trained and experienced research midwife (PH) from the PNG Institute of Medical Research (PNGIMR) and overseen by the principle investigator for the study (LV).

The Eastern Highlands Provincial hospital is the referral hospital for the Eastern Highlands Province, which has an estimated population of 540,000. Two recent studies have been undertaken at the hospital: one identified that 60% of the 29 maternal deaths that occurred over a 40 month retrospective period were attributable to complications of unsafe abortion [26]; the second identified that the majority of women presenting for post abortion care had used misoprostol to end unwanted pregnancies [29].

Over the six month study period we sought to identify all women admitted to the hospital with suspected or confirmed abortion, including both spontaneous and induced abortion. Women were identified through daily review of available admission records at the emergency department, out-patient department, well woman clinic and the obstetrics and gynaecology ward. Inclusion criteria included women admitted with: excessive vaginal bleeding; lower abdominal pain with vaginal discharge/bleeding; fever with vaginal bleeding/discharge and/or; foreign body in-uteri or pelvic injury. In line with the PNG National Department of Health guidelines, abortion was defined as vaginal bleeding before 20 weeks gestation or fetal weight of less than 500grams. Women presenting after 20 weeks gestation were included in the study if they specifically indicated interference with the pregnancy.

### Semi-structured interviews

Following identification of women meeting the inclusion criteria, women were approached by the research midwife who described the nature of the study. For those willing to participate, informed consent procedures were completed prior to completion of a study specific case note record form. Data from this aspect of the study is presented elsewhere [31]. During the consent procedure for the case record form, women were also asked if they were prepared to participate in a semi-structured interview. For those willing to participate in an audio -recorded interview with the research midwife, separate consent was gained.

Semi-structured interviews were included to ensure all cases of induced abortion were identified, whether they had been revealed as such to hospital staff at the time of admission.

We sought to identify women’s reasons for seeking hospital level care, their reaction to the pregnancy and their feelings in relation to the pregnancy loss. Questions in the semi-structured interviews included:

- Can you tell me about why you came to the hospital?
- Can you tell me your story about how the pregnancy ended?
- Do you know why that may have happened?
- How did you feel when the pregnancy ended?

Forty four women participated in the semi-structured interviews of whom 21 had reported that they induced their abortion during the initial hospital admission consultation. As a result of conducting semi-structured interviews an additional four women not reporting any interference with their pregnancy during the hospital admission process disclosed to the research midwife that they had indeed interfered with their pregnancy.

### In-depth interviews

All women identified as having had an induced abortion, either through the case note review or semi-structured interview, were invited by the research midwife to participate in a further in-depth interview to gain additional insight into the individual experiences of these women, including why and how they aborted, and their experiences and perceptions of the health care they received following presentation to hospital. Following informed consent procedures, we used an interview guide to undertake eight in-depth interviews.

### Key informant interviews

All key informants were health care workers and were purposively selected due to their position within their work place. They worked either at the hospital or at local non-government organisations providing sexual and reproductive health services. Despite initial interest in the study, four health care workers declined to participate. In depth interviews were undertaken with eight key informants, using an interview guide, in which they were asked open questions about their experiences of women accessing abortion and post abortion care services. Among the eight key informants, six were from the Eastern Highlands Provincial hospital; four from the ward and two from the accident and emergency department. The remaining two informants were from different NGOs based in Goroka. Seven of the informants were women and six were trained as midwives, including the one male informant. All informants had extensive experience working in both the government and church health services and non-government organisations for between 14 and 36 years.

Interviews with women were undertaken in either *Tok Pisin* (a local *lingua franca*) or English, as preferred by the individual woman. All key informant interviews were undertaken in English. Both the semi-structured and in-depth interview guides were piloted prior to the start of the study. All interviews were undertaken by the research midwife who is trained and experienced in undertaking such interviews.

### Data analysis

All semi-structured interviews were transcribed and translated, where necessary, by the research midwife and reviewed and discussed with the principle investigator to identify additional cases of induced abortion not identified through the hospital admission records. In-depth interviews were transcribed and translated by one member of the research team at the PNGIMR. Transcripts were reviewed by two members of the research team (LV, AK-H) and through a qualitative content analysis approach [32] using continuous comparison an initial coding framework was developed. During the course of analysis, this coding framework was developed and modified as new themes emerged. All transcripts were managed using NVivo9, a qualitative software management programme.

### Ethical considerations

This research was approved by the Institutional Review Board of the PNGIMR (IRB 1201), the Medical Research Advisory Committee (MRAC 11.32), PNG and the University of Queensland Human Ethics Committee in Australia (LV080312). Written consent was obtained from all participants for case note review, semi-structured and in-depth interviews. To ensure anonymity all women participating in the semi structured and in-depth interviews were assigned a pseudonym. To ensure anonymity all key informants were assigned a pseudonym and only their place of work (hospital or NGO) is noted, not their position.

### Findings

Over the six-month study period we identified 129 women who met the inclusion criteria. All women were identified through the ward admission book at the obstetric and gynaecology ward. We positively identified that 92% (119/129) of these women were admitted following a spontaneous or induced abortion. Twenty eight women (28/119; 24%) were admitted following unsafe, induced abortion. Most women (21/28; 75%) reported an induced abortion at the time of admission. Five women (5/28; 18%) had clinical signs that an induced abortion had taken place, two of whom did disclose interference with the pregnancy during the semi-structured interview. Two women (2/28; 7%) who disclosed during their semi structured interview that they had induced their abortion had no clinical signs that the abortion had been induced.

This paper describes themes that emerged during the analysis process. These have been grouped according to the following categories: reasons given for ending the pregnancy; abortion methods used; seeking post abortion care and reflections post abortion.

### Reasons given for ending the pregnancy

Women’s reasons for deciding to end their pregnancy related to the notion of “readiness” for a baby, or related to family or relationship issues.

#### Not ready: Jeopardising a woman’s education

Among younger and single women, many felt they were not ‘ready’ for a baby, in particular it was understood that the pregnancy and a baby would interfere with their education as Nema explains:

“When I told him (boyfriend) … he told [said] me that we were both mad and we are not ready to make a baby and we are not ready to get married… we both didn’t want to leave school. We both didn’t want to have a baby”.

Nema, single, 15–19 years, grade 8 student.

Education is highly valued in PNG and represents a considerable financial investment by a family. The opportunity for secondary education is considered as a means of social mobility. Most families support themselves through subsistence agriculture with few opportunities for wage earning. There is an expectation that children who receive secondary or higher education will be able to secure employment and help support their families and communities through their wages. For young women an education also means better marriage opportunities and increased bride-price (money paid to the woman’s family upon marriage by the groom and his family). In PNG, students studying at school or university are frequently advised by the educational institute to leave school during a pregnancy, with many educational facilities having policies which state a pregnant student cannot be in attendance. Pregnancy therefore threatens a woman’s and her family’s opportunity for social and economic advancement through education.

This sense of a lack of readiness and desire to continue their education was combined with fear and worry about disappointing their family and of bringing shame or embarrassment to their families for being pregnant while still a student or unmarried. Key informants also stated that young girls also feared their parents, as Jay mentions:

“…when they miss their periods they know that they are pregnant… they want it out as soon as possible so, how they go about to get this thing out of them, they go to the extreme…they are desperate to get it out, the young girls they are scared of their parents….”.

Jay, HCW, EHP hospital.

Partly, this fear arose out of knowledge of the financial outlay and sacrifices many families had made towards their education, as Noreen describes:

“As for myself, I thought I must not have this baby, I’m still in school….my family [have spent] a lot of money on school fees and I didn’t think of this and I did that…… I want[ed] to remove it”.

Noreen, 20 years, grade 8 student.

In other cases health workers stated that parents actively sought terminations of pregnancy for their daughters so they could continue their studies. In such cases it was not always clear whether the parents were forcing the young woman to terminate the pregnancy, as Linda described:

“Parents come here and ask “Please is there any way [to end a pregnancy], my daughter is pregnant [and] she needs to continue on with her studies.”…”

Lilian, HCW, EHP hospital.

Although some women were certain that they wished to terminate their pregnancy, others described indecision, resorting to abortion due to fear of the perceived and actual reactions of their families, as Isabella explains:

“….. [I] thought about keeping the baby, however I considered my family, that my father will get cross with me…. I was afraid and [I] made my decision [to have an abortion]”.

Isabella, 22 years, 3rd year university student

#### Gender based violence

There are high rates of gender based violence in PNG [22] but frequently it remains a secretive and shameful topic. One woman in our study presented to hospital reporting an induced abortion, the abortion occurring following physical violence from her husband. No women in our study reported their pregnancy being the result of forced sex, although we did not explicitly ask about this during the interviews. However, as in the case above, there were indications of coerced abortions. In one case, a housewife explained how she was excited at being pregnant again, however her husband did not want the baby and he took his wife to a health care worker himself to ensure an abortion was undertaken:

“My husband brought me to see a relative at the hospital….he did not want the baby so he brought me …..[to get an abortion]”.

Mary, 30–34 years, housewife.

#### Relationship problems

The dynamics of power within their relationship with their husbands was another prominent theme in married women’s discussion of the reasons for their induced abortions. In some cases women explained that their husbands were having extra marital affairs and hence they did not wish to bring another child into that relationship. Rose undertook an abortion as a means of punishing her husband:

“I was happy that I was pregnant but realised my husband was having [an] affair with another woman so I tried ending pregnancy by squeezing my abdomen”.

Rose, 25–29 years, housewife.

#### Cultural beliefs

In the Eastern Highlands Province, as in other settings in PNG, sexual abstinence during breast feeding is understood as a means to prolong breast feeding of the infant to ensure good nutrition for the infant [33]. To be breast feeding while pregnant reveals lack of adherence to this tradition and therefore brings shame to the couple, in addition to which it is felt that the breast milk is not as nutritious for the infant, due to the growing fetus, as Annemarie describes:

“…my child is still an infant and he’s still breastfeeding… if I breast feed him, he will be malnourished because there’s another baby in the womb so, I thought I must remove [abort] this baby, so I removed it”.

Annemarie married, 20–24 years, household duties.

Sorcery, spiritual beliefs and witchcraft are widely believed and spoken about in terms of causes of illness in many cultures within PNG [34] and may be accepted in many communities as a credible explanation for such misfortunes as an abortion. An explanation of sorcery and witchcraft may reposition a woman who aborts from being defined as a perpetrator of a criminal act to a victim. Interference with the pregnancy as a result of witchcraft and evil spirits, directed towards them from another family member was identified during our study, as Elisabeth explains:

“…. I was lifted by spirits and thrown away outside the house by witches two times…then I was hit on the back…. my husband found me outside with blood running like water….”

Elisabeth; 16 weeks gestation, planned pregnancy.

#### Assistance in acquiring an abortion

Usually the person to whom the pregnancy was disclosed to was involved in helping to find the means to end the pregnancy. In some situations that person was the boyfriend, as Noreen describes:

“……he said to me, "I don’t want you to do that (be pregnant), I have a lot of friends so I will get this Cytotec and come and give you and you will end this pregnancy”…. I was happy that he came and gave it to me and I ended this pregnancy…..”

Noreen, 20 years, grade 8 student.

Other women however did not consult with anyone else and acted alone. Kate clearly identifies her agency in acquiring an induced abortion in her following statement:

“… I alone, I myself made my decision and I went and asked around and found it….. my husband does not know…… I went and got the medicine and I drank”.

Kate, separated, 15–19 years, subsistence farmer.

Nema also describes how her boyfriend’s sister-in-law helped, wanting to protect the young couple from unnecessary gossip and information getting back to the young girl’s family:

“My thoughts were to abort it and forget…my boyfriend also said that…He said he would find a way for us and our people [family] would not know…she [the boyfriend’s sister-in-law], told me, “We (the boyfriend and sister in law) will come to town… if our people saw both of you [Nema and her boyfriend] it wouldn’t be good… I will get [your boyfriend] and both of us will go and find a way to get help”. I stayed in the village, that woman [boyfriend’s sister-in-law] bought it [tablets from the pharmacy] and gave [it to] him [boyfriend] and he came and gave [it to] me”.

Nema, single, 15–19 years, grade 8 student.

### Methods of abortion used

A range of methods to end the pregnancy were described, including traditional herbs and physical means, however most women used misoprostol. Key informants mentioned how traditional methods, including the use of herbs have been used for many years in the community setting. While some informants suggested that traditional herbs and physical means continue to be used, others described an increase in women presenting to hospital following the use of misoprostol. Some believe that health care workers are involved in ending an unwanted pregnancy, with women gaining access to misoprostol through prescriptions. There was also some feeling that health care workers s in some health facilities were providing abortions, although the abortion methods and techniques were not discussed.

#### Misoprostol

Women who reported using misoprostol to end their pregnancy took between two and five tablets and both oral and vaginal routes of administration were described. The misoprostol was obtained through a pharmacy and frequently a family member, friend or boyfriend was involved in procuring the tablets. The purchase was not always straight forward, as Nema explains:

“They themselves [chemist] have stopped selling to the public [meaning not displayed on the shelves]. But there are relatives…they gave it to her [referring to a friend]”.

Nema, single, 15–19 years, grade 8 student.

Lucinda explained how she has seen a change in abortion methods used with misoprostol becoming more widely recognised as a method of abortion:

“By my observation…..it’s changed, now they are more to [using] Misoprostol….. it’s easier than trying to use these irons and sticks, and normally people in the village too they come, they ask for this Misoprostol. Like the educated people living in the village, they’ve done their grade 10, grade 12 and they are in the village, they come and ask….female [relatives] for it. Like if a mother notices that her child is expecting, she’ll come and ask on behalf of her daughter”.

Lucinda, HCW, EHP hospital.

Confirming the suspicions of some of the health care workers, there were reports of women obtaining misoprostol through health care workers at hospitals outside of Goroka (the capital of EHP), and through a prescription obtained from another hospital, as Monalisa and Tina explain:

“I took tablets. Women who used it told me, they bought it from this man [at a health facility] so I went and got it directly. He put it [in] and I came… I removed it [the fetus]….”.

Monalisa, separated, 32 years, housewife.

“I came to [the hospital] and I did a pregnancy test and it was positive so they prescribed a medicine for me to take, and I went to the chemist and I got the medicine…She [the nurse] said, “go to the pharmacy because at the hospital we do not supply this medicine….”.

Tina, unmarried, 20 years, grade 11 student.

Women were able to recall quite clearly the instructions provided when buying the misoprostol, however, for many the instructions and advice was incorrect, as Kate describes:

“… I bought it … they told me how to use it … I went… I drank 2 [and] I inserted 2 in the vagina…. I waited and then I felt a bit alright and then, it [the fetus] came out”.

Kate, separated, 19 years; induced at less than 12 weeks.

This incorrect messaging and consequences of incorrect dosage of misoprostol was highlighted by the key informants, as Jay describes:

“She went and she bought some drugs from somebody saying they were a doctor from the hospital [and] this girl said this guy gave her six tablets, and he instructed her to put it up her vagina and it will help her to contract and she will abort the baby. But this dose was too much for her, she came and she was in so much pain, she was screaming and she was yelling and we told [asked] her, “what did you do?” and then she said, “oh someone gave me something and I put it [in] and this is what happened….”

Jay, HCW, EHP hospital

The only women who reported a dose and route correct for their gestation were those who received their misoprostol from health care workers.

For those women who reported the costs involved in purchasing the misoprostol, none expressed difficulty finding the money, even though many of the women were students or housewives with very little income. Monalisa describes how she had to find K200 (US$ 75) to pay a health care worker for two tablets:

“….it’s expensive, they usually charge for them a lot of money, but as for myself, I promised that I will pay half…. I went and gave him K40.00 together with a *bilum* [traditional woven bag of high value] …I promised I will not hide, I will go and pay for [the rest of]it ….”.

Monalisa, separated, 32 years, housewife.

Frequently the cost was met by family member’s, or the boyfriend and could be negotiated, as Nema describes:

“…he told me that they charged K200.00 but that woman [boyfriend’s sister-in-law] made friends with them and she said “they are school students who came to me with this problem,” …she said “I have K130.00” and they helped her”.

Nema single, 19 years, grade 8 student.

#### Traditional herbs

The use of traditional herbs, in particular tree bark or grasses chewed up and swallowed or squeezed to make a juice were described by both women and key informants. Following their use women reported abdominal cramps and vomiting before expulsion of the uterine contents, as Velma describes:

“[I] ate some herbs- grass, put salt and ate [the] soft part, squeezed the green plant and put salt on and the water drip into [my] mouth and I swallowed it. [I was helped by] a woman in the village who knows that…. for K20.00. [I] felt pain generalized all over the body, headache, backache and then [I] gave birth to a baby boy- [fetus], and he made a little noise then [I] cut the cord”.

Velma, married, 16 weeks at induced abortion.

Key informants also described traditional methods as an effective means of ending a pregnancy, as Katherine explains:

“When I interview them I find that they were using some tree barks, and some grass, which they locally use to induce abortion. Traditional methods…. grass… they just pick the grass and chew it and swallow to induce the abortion, [same with] the bark of the tree”.

Katherine, HCW, NGO Goroka.

However, there was some concern among the key informants that these traditional methods can be ineffective, leaving women vulnerable to post abortion complications, as Frances explains:

“It takes 24 hours for this thing to work… in the past, those people that were using [preparing and administering] the barks of a tree were elderly men - that [what] I’ve seen, where I come from. Some [women] they try those things and if it doesn’t work, then they go for some [other] induced abortion…But I’ve witnessed that, the bark of a tree works. I’ve seen [it] and I’ve witnessed [it], It’s very effective…it terminates the pregnancy but…. it doesn’t clear the uterus, it doesn’t expel everything out from the uterus so, there are chances that the mother will have complications from that”.

Frances, HCW, NGO, Goroka.

A few women combined the traditional methods of abortion with modern methods. Monalisa describes how she initially sought traditional abortifacients, but when these did not work she resorted to misoprostol. As in her case, trying various means to abort may result in delays, increasing the risks to women as the gestational age increases:

“I said I’ll try in the village, get ginger and those things and help myself …. they usually plant it differently, the ginger …for aborting babies… I gave him K10.00….he [the medicine man] brought it, spoke [some words over it]…brought it, still talking and poking it [piercing the stem of ginger with a needle] but when he pulled it out it was strong, and he said…“it’s strong”- it means that he is not able to remove it [fetus], so he said, “that’s alright, leave it”. I myself I don’t believe much about this thing in the village, when I felt I did this… I saw it I said “ah stupid….”. Those things to abort a child, tree bark or that kind of thing…I said I must go to the hospital…so I came.

Monalisa, separated, 32 years, housewife [induced at 5 months using misoprostol].

#### Physical means

Squeezing or tying a rope around the abdomen, undertaking excessive exercise, running over mountains and jumping over streams as a means to end the pregnancy were also described. Annemarie explained how she waited until she knew the pregnancy would be far enough progressed to enable her to exert enough force on her lower abdomen to interrupt the pregnancy:

“…I went past 3 months and I squeezed my abdomen and I killed one [the] baby boy and I removed it … I used my hand, myself and squeezed my abdomen 3 times I tried to remove it [abort] and the 4th time I removed it. I allowed the baby to grow big then I squeezed it [abdomen] and removed it. If it was small and I removed [aborted] it will die inside the womb and it will fester [decay]inside so I was a little scared and I removed it….”

Annemarie, married, 16 weeks at abortion.

One young woman, widowed after a tribal fight in her community described how she turned to her sister for advice on ending her pregnancy, inserting a stick into her vagina to end the pregnancy at eight weeks gestation:

“[My] sister informed me about [using] the plant [stick] and I went to [the] bush and removed it [the fetus]”.

Sue, 19 years, widow.

Reflecting many of the methods reported from women in this study, key informants revealed their experiences from both the community and professionally, having witnessed physical means to end a pregnancy, as Lilian and Okaps describe:

“….to induce the abortion, some they do it themselves [these] women…get rid of the pregnancy by themselves, they do all sorts of things…they push sharp instruments into the cervix or into the uterus, and we’ve witnessed and seen trauma, infected, they come in very septic and some…they take some herbs or they drink strong coffee or alcohol they go into all these [methods] they think they can consume this one to destroy the pregnancy, and some they step on their abdomen, step on their abdomen and do all these things to force the pregnancy out”.

Lilian, HCW, EHP hospital.

“ I saw them, the mothers would sit down on top of the abdomen of the young girl and they crush and abort the baby”.

Okaps, male HCW, EHP hospital.

### Seeking care post abortion

Key informants spoke of the secrecy surrounding induced abortion, which contravenes social, cultural and Christian norms in PNG and evokes fear of prosecution among women. The issue of not wanting to disclose an induced abortion was highlighted by the key informants who recognised that often women presenting to hospital do not disclose having induced an abortion, which is identified only upon clinical examination, as Cinta mentioned:

“When women, from [their] history they present we collect information and at times when you are doing speculum examination, you can see that if it is criminal abortion like, you’ll see objects like stick or a piece of iron rod or something, you can see, the cervical os and the cervix inside is rough and rugged….and it’s bleeding from the tear, so you can tell that, it’s criminal abortion which has been induced with instruments….”

Cinta, HCW, EHP hospital.

Despite the implications involved and the stigma and secrecy surrounding abortion, the women in this study presented to hospital because they had concerns about complications and the consequent implications on their health, as Tina explains:

“I was a little scared because, I heard that this is illegal, it's an illegal abortion. I was a little scared but I knew that if I came to the hospital I will get help ….”.

Tina, unmarried, 20 years, grade 11 student.

However, frequently women delayed seeking care post abortion, many presenting for hospital level care between six days and up to four weeks after the abortion had taken place [31]. For many the delay was because the abortion had taken place without the knowledge of those who the women lived closely with, seeking care meant disclosing what had transpired, as Noreen describes:

“I thought that if I don’t come to the hospital and get help, I remain in the house I will get worse and die….I would get worse if I didn’t tell my family. That’s why when I told my family they helped me come to the hospital”.

Noreen, 20 years, grade 8 student.

Women described a number of symptoms that triggered them to seek care at the hospital. While women expected to see vaginal bleeding, many became concerned when this went on for longer than they expected, they saw blood clots or when they experienced other symptoms such as feeling dizzy or abdominal and back pain. Some women felt their symptoms were so severe they feared they may die if they did not receive health care. A few spoke of the need to come to the hospital in order to be “cleaned”, to ensure no products of conception remained. For many of the women, once they had disclosed their situation to the family a vehicle was hired or made available to bring the women into the hospital. Some arrived by a local bus, and others were brought in by ambulance after presenting to their nearest health facility.

### Reflections post abortion

A number of the women spoke about their feelings relating to ending their pregnancy. While most felt relieved that they were no longer pregnant, a few related feelings of grief and spoke of regret for what they had done. Annemarie describes feeling relieved, managing the situation as she felt appropriate:

“Hmm when he [the fetus] came out straight, I was thinking my [breast feeding] infant will drink good breast milk and will have more strength and he will be fine so I’m happy that I removed it…. We wrapped it [the fetus] with a napkin and I covered him then I buried him inside a hole”.

Annemarie, aborted at 16 weeks.

In contrast Noreen – a young, single woman with no previous pregnancy history describes her feelings of guilt on aborting her fetus at 12 weeks gestation:

“I thought back again why [did] I abort this child and I wasn’t happy. When I removed it, I noticed the child had formed already…. and I thought back again why did I abort it, I should have kept it”.

Noreen, 20 years old, student

For some women the grief and loss was made harder by a lack of empathy from the health care workers at the hospital:

“..I even felt sorry for the little innocent [fetus]…I felt shy, guilty…and even sorrow…he [the doctor] was really cross with me….”.

Beth, aborted at 11 weeks

## Discussion

We identified 28 women admitted to hospital following an induced abortion. Women’s reasons for seeking an abortion related to: a lack of “readiness” and poor timing of the pregnancy, especially with relation to women’s education; not wanting to cause shame or embarrassment to themselves or their family; relationship problems and some cultural beliefs.

Poor timing of pregnancy, including being young and unmarried and wanting to space children is reported from a number of settings as a reason for ending an unplanned pregnancy [13,35]. In addition to these reasons, out of a fear of a missed opportunity for education and all that that could mean for the individuals and their families, coupled with a fear of responses from the family, it is not perhaps surprising that the women in our study resorted to induced abortions. In PNG 5% of females finish education early because of pregnancy and marriage, in the Highlands region the rate is 9% [17]. As the average age of sexual debut in PNG is 18.7 years for females and the average age at the birth of a first child is 20.8 years [23], it is not unexpected that many school and tertiary students are experiencing unplanned pregnancies.

Many of the women reported the use of misoprostol to end the unwanted pregnancy, a medical method increasingly being used to end unwanted pregnancy in many developing countries [3,7,10,29,36,37], including earlier reports from PNG [29]. While the use of misoprostol is associated with less severe outcomes and morbidity, compared to the use of substances and physical methods [4,7,8], it is only safer when factors of gestation and correct dose are followed [38,39]. Lack of adequate supervision from a skilled health care person [38] and following a sub-optimal misoprostol regime, can lead to several days of hospitalisation [40]. Women in our study reporting the use of misoprostol described doses and regimes inaccurate for their stated gestation, and lack of supervision from an appropriate health care provider was noted. Given the availability of misoprostol in this setting, it is also possible that other women may have undertaken abortions in the first trimester with less severe or no complications, thus not requiring hospital level care and not identified in this study.

Highlighted by both the key informants and women participating in this study, the use of physical and mechanical methods to end an unwanted pregnancy are still used by some women. However, the increasing availability of misoprostol is perhaps providing an alternative method for women, findings identified elsewhere [4,7,8]. As reported from other settings [4,35], women in our setting also reported the use of herbal remedies to induce abortion. The large evergreen tree, *Alstonia scholaris* is distributed throughout PNG and is recognised for a number of traditional uses, including chewing the leaves as an oral contraceptive, and ingestion of the dried bark sap to induce abortion [41]. While in some communities in PNG there is a general knowledge of plant preparations, in some situations there may be the need to enlist the more specialized knowledge of a traditional healer or, in some situations a sorcerer. In our study some of the women using traditional, herbal methods to end their pregnancy enlisted the help of older village women; seeking assistance from a “medicine man” who used sorcery in anattempt to interrupt an unwanted pregnancy.

While grounds on which abortion can be legally performed has broadened in many developing countries, in countries where it remains illegal, abortions frequently continue to take place in unsafe circumstances [6]. In PNG, as in other settings, the factors that hinder access to safe abortion also play a role in women accessing health services for post abortion care, following an unsafe abortion [2,42]. This study highlights that women fear repercussions from both health care workers and the legal framework surrounding abortion practices, findings reflected in other settings [2,7]. Also noted through this study was the use by health care workers of the term “criminal abortion” to describe and induced abortion, perhaps highlighting the attitude of health care workers towards abortion in this setting. The fear of presenting for hospital level care led to delays in seeking assistance, both from family members and the formal health systems with many women only reported seeking health care when they felt their lives were at risk.

As described elsewhere, where induced abortion is restricted or inaccessible, identifying and reporting abortion is difficult [2,11]. Even in settings where abortion is legal, it may be under reported or reported as spontaneous, especially when it has occurred outside of a legal framework [2]. While most women reported interference with their pregnancy at their admission consultation, some only identified as an induced abortion during their semi structured interview.

This study describes only women presenting for hospital level care. One limitation of such a hospital based study is that it only captures those women able to access a health facility or with a morbidity so severe that they present for hospital level care. Frequently the young, the poor and those living in the more remote areas are at greatest risk of not reaching health services for post abortion care [7,42,43]. In addition, women experiencing a complete abortion, those with less severe morbidity, and those who undertake an abortion resulting in a maternal death remain unaccounted for in the community, therefore never forming part of any official statistics. Only capturing those who reach the hospital only represent a sub-population of all women in this setting undertaking an induced abortion.

## Conclusion

This descriptive study provides insight into an area of maternal health not sufficiently in PNG. As in many developing countries, women in PNG are vulnerable to unplanned pregnancies. In the absence of adequate family planning services, particularly for the young and those in still in education, women are resorting to unsafe means to end an unwanted pregnancy. In addition their lives are put further at risk from delayed health care seeking due to fear of repercussions from both their family, health care workers and the legal framework surrounding abortion. Review of and improved access to safe abortion services together with and dissemination of the legality of induced abortion in PNG could help in reducing the burden of maternal mortality and morbidity from unsafe, induced abortions in this setting.

## Abbreviations

CPR: Contraceptive prevalence rate
DHS: Demographic and Health Survey
EHP: Eastern Highlands Province
HCW: Health Care Worker
IDI: In depth Interview
IRB: Internal Review Board
MMR: Maternal Mortality Ratio
MRAC: Medical Research Advisory Committee
NDoH: National Department of Health
NHIS: National health information System
NGO: Non-Government Organisation
PNG: Papua New Guinea
PNGIMR: Papua New Guinea Institute of Medical Research
PAC: Post abortion care
SSI: Semi structured interview
TFR: Total Fertility Rate
USAID: United States Agency for International Development
WHO: World Health Organization
